# Accelerating materials property predictions using machine learning

**DOI:** 10.1038/srep02810

**Published:** 2013-09-30

**Authors:** Ghanshyam Pilania, Chenchen Wang, Xun Jiang, Sanguthevar Rajasekaran, Ramamurthy Ramprasad

**Affiliations:** 1 Department of Materials Science and Engineering, University of Connecticut, 97 North Eagleville Road, Storrs, Connecticut 06269; 2 Department of Statistics, University of Connecticut, 215 Glenbrook Road, Storrs, Connecticut 06269; 3 Department of Computer Science and Engineering, University of Connecticut, 371 Fairfield Road, Storrs, Connecticut 06269

## Abstract

The materials discovery process can be significantly expedited and simplified if we can learn effectively from available knowledge and data. In the present contribution, we show that efficient and accurate prediction of a diverse set of properties of material systems is possible by employing machine (or statistical) learning methods trained on quantum mechanical computations in combination with the notions of chemical similarity. Using a family of one-dimensional chain systems, we present a general formalism that allows us to discover decision rules that establish a mapping between easily accessible attributes of a system and its properties. It is shown that fingerprints based on either chemo-structural (compositional and configurational information) or the electronic charge density distribution can be used to make ultra-fast, yet accurate, property predictions. Harnessing such learning paradigms extends recent efforts to systematically explore and mine vast chemical spaces, and can significantly accelerate the discovery of new application-specific materials.

Owing to the staggering compositional and configurational degrees of freedom possible in materials, it is fair to assume that the chemical space of even a restricted subclass of materials (say, involving just two elements) is far from being exhausted, and an enormous number of new materials with useful properties are yet to be discovered. Given this formidable chemical landscape, a fundamental bottleneck to an efficient materials discovery process is the lack of suitable methods to rapidly *and* accurately predict the properties of a vast array (within a subclass) of new yet-to-be-synthesized materials. The standard approaches adopted thus far involve either expensive and lengthy Edisonian synthesis-testing experimental cycles, or laborious and time-intensive computations, performed on a case-by-case manner. Moreover, neither of these approaches is able to readily unearth Hume-Rothery-like “hidden” semi-empirical rules that govern materials behavior.

The present contribution, aimed at materials property predictions, falls under a radically different paradigm^1^,^2^, namely, machine (or statistical) learning—a topic central to network theory^3^, cognitive game theory^4^,^5^, pattern recognition^6^,^7^,^8^, artificial intelligence^9^,^10^, and event forecasting^11^. We show that such learning methods may be used to establish a mapping between a suitable representation of a material (i.e., its ‘fingerprint’ or its ‘profile’) and *any* or *all* of its properties using known historic, or intentionally generated, data. The material fingerprint or profile can be coarse-level chemo-structural descriptors, or something as fundamental as the electronic charge density, both of which are explored here. Subsequently, once the profile 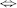 property mapping has been established, the properties of a vast number of new materials within the same subclass may then be directly predicted (and correlations between properties may be unearthed) at negligible computational cost, thereby completely by-passing the conventional laborious approaches towards material property determination alluded to above. In its most simplified form, this scheme is inspired by the intuition that (dis)similar materials will have (dis)similar properties. Needless to say, training of this intuition requires a critical amount of prior diverse information/results^12^,^13^,^14^,^15^,^16^ and robust learning devices^12^,^17^,^18^,^19^,^20^,^21^,^22^.

The central problem in learning approaches is to come up with decision rules that will allow us to establish a mapping between measurable (and easily accessible) attributes of a system and its properties. Quantum mechanics (here employed within the framework of density functional theory, DFT)^23^,^24^, provides us with such a decision rule that connects the wave function (or charge density) with properties via the Schrödinger's (or the Kohn-Sham) equation. Here, we hope to replace the rather cumbersome rule based on the Schrödinger's or Kohn-Sham equation with a module based on similarity-based machine learning. The essential ingredients of the proposed scheme is captured schematically in Figure 1.

## Results

**Figure 1:**
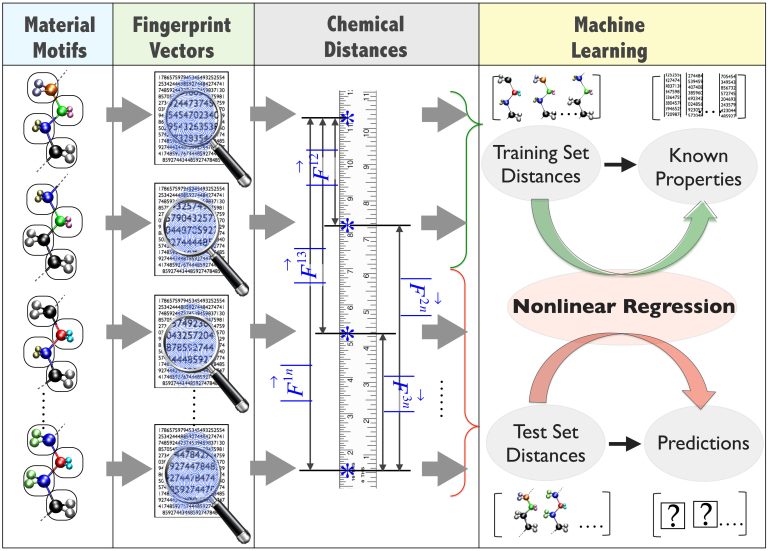
The machine (or statistical) learning methodology. First, material motifs within a class are reduced to numerical fingerprint vectors. Next, a suitable measure of chemical (dis)similarity, or chemical distance, is used within a learning scheme—in this case, kernel ridge regression—to map the distances to properties.

The ideal testing ground for such a paradigm is a case where a parent material is made to undergo systematic chemical and/or configurational variations, for which controlled initial training and test data can be generated. In the present investigation, we consider infinite polymer chains—quasi 1-d *material motifs* (Figure 1)—with their building blocks drawn from a pool of the following seven possibilities: CH_2_, SiF_2_, SiCl_2_, GeF_2_, GeCl_2_, SnF_2_, and SnCl_2_. Setting all the building blocks of a chain to be CH_2_ leads to polyethylene (PE), a common, inexpensive polymeric insulator. The rationale for introducing the other Group IV halides is to interrogate the beneficial effects (if any) these blocks may have on various properties when introduced in a base polymer such as PE. The properties that we will focus on include: the atomization energy, the formation energy, the lattice constant, the spring constant, the band gap, the electron affinity, and the optical and static components of the dielectric constant. The initial dataset for 175 such material motifs containing 4 building blocks per repeat unit was generated using DFT.

The first step in the mapping process prescribed in the panels of Figure 1 is to reduce each material system under inquiry to a string of numbers—we refer to this string as the *fingerprint vector*. For the specific case under consideration here, namely, polymeric chains composed of seven possible building blocks, the following coarse-level chemo-structural fingerprint vector was considered first: |*f*_1_, …, *f*_6_, *g*_1_, …, *g*_7_, *h*_1_, …, *h*_7_〉, where *f_i_*, *g_i_* and *h_i_* are, respectively, the number of building blocks of type *i*, number of *i* − *i* pairs, and number of *i* − *i* − *i* triplets, normalized to total number of units (note that *f*_7_ is missing in above vector as it is not an independent quantity owing to the relation: 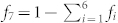). One may generalize the above vector to include all possible *i* − *j* pairs, *i* − *j* − *k* triplets, *i* − *j* − *k* − *l* quadruplets, etc., but such extensions were found to be unnecessary as the chosen 20-component vector was able to satisfactorily codify the information content of the polymeric chains.

Next, a suitable measure of *chemical distance* is defined to allow for a quantification of the degree of (dis)similarity between any two fingerprint vectors. Consider two systems *a* and *b* with fingerprint vectors 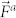 and 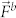. The similarity of the two vectors may be measured in many ways, e.g., using the Euclidean norm of the difference between the two vectors, 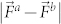, or the dot product of the two vectors 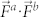. In the present work, we use the former, which we refer to as 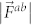 (Figure 1). Clearly, if 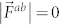, materials *a* and *b* are equivalent (insofar as we can conclude based on the fingerprint vectors), and their property values *P^a^* and *P^b^* are the same. When 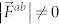, materials *a* and *b* are not equivalent, and *P^a^* − *P^b^* is not necessarily zero, and depends on 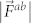. This observation may be formally quantified when we have a prior materials-property dataset, in which case we can determine the parametric dependence of the property values on 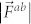.

**Figure 2:**
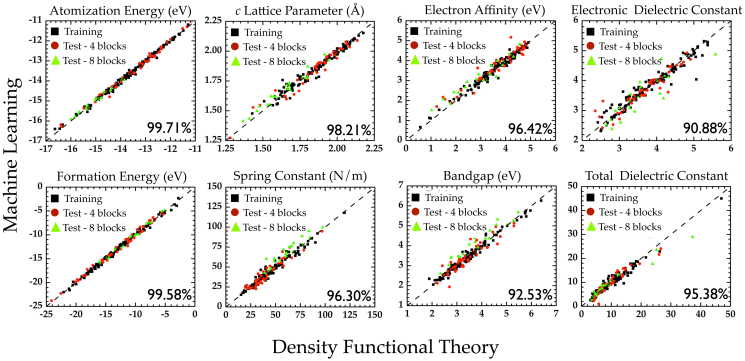
Learning performance of chemo-structural fingerprint vectors. Parity plots comparing property values computed using DFT against predictions made using learning algorithms trained using chemo-structural fingerprint vectors. Pearson's correlation index is indicated in each of the panels to quantify the agreement between the two schemes.

In the present work, we apply the *machine learning* algorithm referred to as kernel ridge regression (KRR)^25^,^26^, to our family of polymeric chains. Technical details on the KRR methodology are provided in the Methods section of the manuscript. As mentioned above, the initial dataset was generated using DFT for systems with repeat units containing 4 distinct building blocks. Of the total 175 such systems, 130 were classified to be in the ‘training’ set (used in the training of the KRR model, Equation (1)), and the remainder in the ‘test’ set. Figure 2 shows the agreement between the predictions of the learning model and the DFT results for the training and the test sets, for each of the 8 properties examined. Furthermore, we considered several chains composed of 8-block repeat units (in addition to the 175 4-block systems), performed DFT computations on these, and compared the DFT predictions of the 8-block systems with those predicted using our learning scheme. As can be seen, the level of agreement between the DFT and the learning schemes is uniformly good for all properties across the 4-block training and test set, as well as the somewhat out-of-sample 8-block test set (regardless of the variance in the property values). Moreover, properties controlled by the local environment (e.g., the lattice parameter), as well as those controlled by nonlocal global effects (e.g., the electronic part of the dielectric constant) are well-captured. We do note that the agreement is most spectacular for the energies than for the other properties (as the former are most well-converged, and the latter are derived or extrapolated properties; see Methods). Overall, the high fidelity nature of the learning predictions is particularly impressive, given that these calculations take a minuscule fraction of the time necessitated by a typical DFT computation.

**Figure 3:**
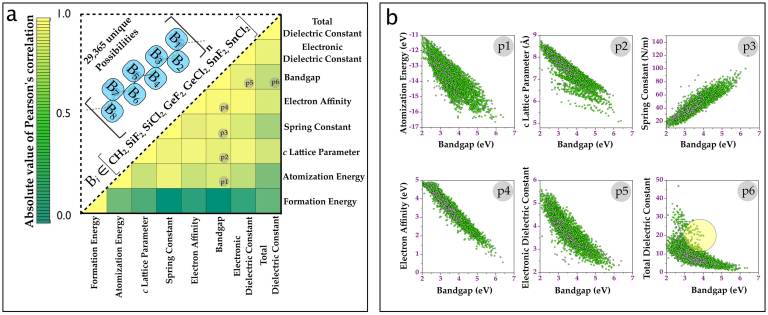
High throughput predictions and correlations from machine learning. (a) The upper triangle presents a schematic of the atomistic model composed of repeat units with 8 building blocks. Populating each of he 8 blocks with one of the seven units leads to 29,365 systems. The matrix in the lower triangle depicts the Pearson's correlation index for each pair of the eight properties of the 8-block systems predicted using machine learning. (b) Panels p1 to p6 show the correlations between the band gap and six properties. The panel labels are also appropriately indexed in (a). The circle in panel p6 indicates systems with a simultaneously large dielectric constant and band gap.

While the favorable agreement between the machine learning and the DFT results for a variety of properties is exciting, in and of itself, the real power of this prediction paradigm lies in the possibility of exploring a *much* larger chemical-configurational space than is practically possible using DFT computations (or laborious experimentation). For instance, merely expanding into a family of 1-d systems with 8-block repeat units leads to 29,365 symmetry unique cases (an extremely small fraction of this class was scrutinized above for validation purposes). Not only can the learning approach make the study of this staggeringly large number of cases possible, it also allows for a search for correlations between properties in a systematic manner. In order to unearth such correlations, we first determined the properties of the 29,365 systems using our machine learning methodology, followed by the estimation of Pearson's correlation coefficient for each pair of properties. The Pearson correlation coefficient (*r*) used to quantify a correlation between two given property datasets {*X_i_*} and {*Y_i_*} for a class of *n* material systems is defined as follows: 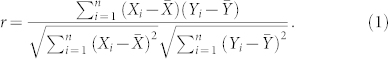
Here, 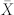 and 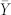 represent the average values of the properties over the respective datasets. Figure 3a shows a matrix of the correlation coefficients, color-coded to allow for immediate identification of pairs of properties that are most correlated.

It can be seen from Figure 3a that the band gap is most strongly correlated with many of the properties. Panels p1–p6 of Figure 3b explicitly show the correlation between the band gap and six of the remaining seven properties. Most notably, the band gap is inversely correlated with the atomization energy (p1), size (p2), electron affinity (p4), and the dielectric constants (p5 and p6), and directly correlated with the spring constant (p3). The relationships captured in panels p1–p3 follow from stability and bond strength arguments. The interesting inverse relationship between the band gap and the electron affinity is a consequence of the uniform shift of the conduction band minimum (due to changes in the band gap) with respect to the vacuum level. The inverse correlation of the band gap with the electronic part of the dielectric constant follows from the quantum mechanical picture of electronic polarization being due to electronic excitations. As no such requirement is expected for the ionic part of the dielectric constant, it is rather surprising that a rough inverse correlation is seen between the total dielectric constant and the band gap, although clear deviations from this inverse behavior can be seen. Finally, we note that the formation energy is uncorrelated with all the other seven properties, including the band gap. This is particularly notable as it is a common tendency to assume that the formation energy (indicative of thermodynamic stability) is inversely correlated with the band gap (indicative of electronic stability).

## Discussion

Correlation diagrams such as the ones in Figure 3b offer a pathway to ‘design’ systems that meet a given set of property requirements. For instance, a search for insulators with high dielectric constant *and* large band gap would lead to those systems that are at the top part of panel p6 of Figure 3b (corresponding to the ‘deviations’ from the inverse correlation alluded to above, and indicated by a circle in panel p6). These are systems that contain 2 or more contiguous SnF_2_ units, but with an overall CH_2_ mole fraction of at least 25%. Such organo-tin systems may be particularly appropriate for applications requiring high-dielectric constant polymers. Furthermore, such diagrams can aid in the extraction of knowledge from data eventually leading to Hume-Rothery-like semi-empirical rules that dictate materials behavior. For instance, the panel p3 reveals a well known correspondence between mechanical strength and chemical stability^27^, while panels p5 and p6 capture an inverse relationship between the dielectric constant and the bandgap, also quite familiar to the semiconductor physics community^28^.

The entire discussion thus far has focused on fingerprint vectors defined in terms of coarse-level chemo-structural descriptors. This brings up a question as to whether other more fundamental quantities may be used as a fingerprint to profile a material. The first Hohenberg-Kohn theorem of DFT^29^ proves that the electronic charge density of a system is a universal descriptor containing the sum total of the information about the system, including all its properties. The shape^30^ and the holographic^31^ electron density theorems constitute further extensions of the original Hohenberg-Kohn theorem. Inspired by these theorems, we propose that machine learning methods may be used to establish a mapping between the electronic charge density and various properties.

**Figure 4:**
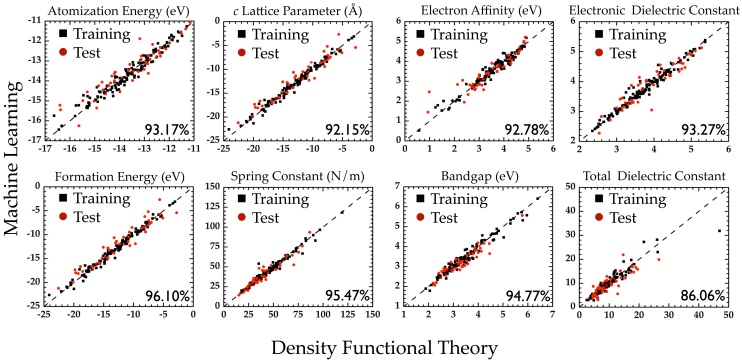
Learning performance of electron charge density-based fingerprint vectors. Parity plots comparing property values computed using DFT against predictions made using learning algorithms trained using electron density-based fingerprint vectors. The Fourier coefficients of the planar-averaged Kohn-Sham charge density are used to construct the fingerprint vector. Pearson's correlation index is indicated in each of the panels to quantify the agreement between the two schemes.

A fundamental issue related to this perspective deals with defining a (dis)similarity criterion that can enable a fair comparison between the charge density of two different systems. Note that any such measure has to be invariant with respect to relative translations and/or rotations of the systems. In the present work, we have employed Fourier coefficients of the 1-d charge density of our systems (averaged along the plane normal to the chain axis). The Fourier coefficients are invariant to translations of the systems along the chain axis, and consideration of the 1-d planar averaged charge density makes the rotational degrees of freedom irrelevant. Figure 4 shows a comparison of the predictions of the learning model based on charge density with the corresponding DFT results. While the agreement between the learning scheme and DFT is not as remarkable as with the chemo-structural fingerprint approach adopted earlier, this can most likely be addressed by the utilization of the actual 3-d charge density. Nevertheless, we believe that the performance of the learning scheme is satisfactory, and heralds the possibility of arriving at a ‘universal’ approach for property predictions solely using the electronic charge density.

A second issue with the charge density based materials profiling relates to determining the charge density in the first place. If indeed a mapping between charge density and the properties can be made for the training set, how do we obtain the charge density of a new system without explicitly performing a DFT computation? We suggest that the ‘atoms in molecules’ concept may be exploited to create a patched-up charge density distribution^32^. Needless to say, barring some studies in the area of atoms and molecules^33^, these concepts are in a state of infancy, and there is much room available for both fundamental developments and innovative applications.

To conclude, we have shown that the efficient *and* accurate prediction of a diverse set of unrelated properties of material systems is possible by combining the notions of chemical (dis)similarity and machine (or statistical) learning methods. Using a family of 1-d chain systems, we have presented a general formalism that allows us to discover decision rules that establish a mapping between easily accessible attributes of a system and its various properties. We have unambiguously shown that simple fingerprint vectors based on either compositional and configurational information, or the electronic charge density distribution, can be used to profile a material and make property predictions at an enormously small cost compared either with quantum mechanical calculations or laborious experimentation. The methodology presented here is of direct relevance in identifying (or screening) undiscovered materials in a targeted class with desired combination of properties in an efficient manner with high fidelity.

## Methods

### First principles computations

The quantum mechanical computations were performed using density functional theory (DFT)^23^,^24^ as implemented in the Vienna *ab initio* software package^34^,^35^. The generalized gradient approximation (GGA) functional parametrized by Perdew, Burke and Ernzerhof (PBE)^36^ to treat the electronic exchange-correlation interaction, the projector augmented wave (PAW)^37^ potentials, and plane-wave basis functions up to a kinetic energy cutoff of 500 eV were employed.

Our 1-d systems were composed of all-trans infinitely long isolated chains containing 4 independent building units in a supercell geometry (with periodic boundary conditions along the axial direction). One CH_2_ unit was always retained in the backbone (to break the extent of *σ*-conjugation along the backbone), and the three other units were drawn from a “pool” of seven possibilities: CH_2_, SiF_2_, SiCl_2_, GeF_2_, GeCl_2_, SnF_2_ and SnCl_2_, in a combinatorial and exhaustive manner. This scheme resulted in 175 symmetry unique systems after accounting for translational periodicity and inversion symmetry. A Monkhorst-Pack *k*-point mesh of 1 × 1 × *k* (with *kc* > 50) was used to produce converged results for a supercell of length *c* Å along the chain direction (*i.e.*, the *z* direction). The supercells were relaxed using a conjugate gradient algorithm until the forces on all atoms were <0.02 eV/Å and the stress component along the *z* direction was <1.0 × 10^−2^ GPa. Sufficiently large grids were used to avoid numerical errors in fast Fourier transforms. A small number of cases involving 8 building units were also performed for validation purposes.

The calculated atomization energies and formation energies are referenced to the isolated atoms and homo-polymer chains of the constituents, respectively. While the lattice parameters, spring constants, band gaps and electron affinities of the systems are readily accessible through DFT computations, the calculations of the optical and static components of the dielectric constant require particular care. The dielectric permittivity of the isolated polymer chains placed in a large supercell were first computed within the density functional perturbation theory (DFPT)^38^,^39^ formalism, which includes contributions from the polymer as well as from the surrounding vacuum region of the supercell. Next, treating the supercell as a vacuum-polymer composite, effective medium theory^40^ was used to estimate the dielectric constant of just the polymer chains using methods described recently^13^,^41^. Table 1 of the Supporting Information contains the DFT computed atomization energies, formation energies, *c* lattice parameters, spring constants, electron affinities, bandgaps, and dielectric permittivities for the 175 symmetry unique polymeric systems.

### Machine learning details

Within the present similarity-based learning model, a property of a system in the test set is given by a sum of weighted Gaussians over the entire training set, as 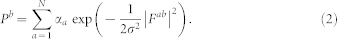
where *a* runs over the systems in the previously known dataset. The coefficients *α_a_*s and the parameter *σ* are obtained by ‘training’ the above form on the systems *a* in the previously known dataset. The training (or learning) process is built on minimizing the expression 
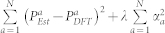
, with 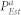 being the estimated property value, 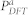 the DFT value, and *λ* a regularization parameter^25^,^26^. The explicit solution to this minimization problem is ***α*** = (***K*** + ***λI***)**^−1^*P_DFT_***, where ***I*** is the identity matrix, and 
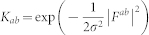
 is the kernel matrix elements of all polymers in the training set. The parameters *λ*, *σ* and *α_a_*s are determined in an inner loop of fivefold cross validation using a logarithmically scaling fine grid.

## Author Contributions

R.R., C.W. and G.P. conceived the statistical learning model, with input from S.R. and X.J. The DFT computations were performed by G.P. The initial implementation of the statistical learning framework was performed by C.W. and extended by G.P. The manuscript was written by G.P., S.R. and R.R.

## Supplementary Material

Supplementary Information Supporting Information for Accelerating materials property predictions using machine learning
