# Supplementary material for: Accelerating materials property predictions using machine learning
Source: Sci Rep. 2013 Sep 30;3:2810. doi: 10.1038/srep02810 (PMC3786293; doi:10.1038/srep02810)
Supplement: Supplementary Information — Supporting Information for Accelerating materials property predictions using machine learning [file srep02810-s1.pdf]

Supporting Information for

Accelerating materials property predictions using machine learning

G. Pilania, C. C. Wang, X. Jiang, S. Rajasekaran, R. Ramprasad*

E-mail: rampi@uconn.edu

Table 1: The DFT computed atomization energies, formation energies, c lattice parameters, spring constants, electron affinities, bandgaps, and dielectric permittivities, both electronic (ε_e^c) and total (ε_{tot}^c) along the chain axis, for the 175 symmetry unique 4-unit polymeric systems. DFT total energies of bulk C, Si, Ge and Sn as well as H_2, F_2 and Cl_2 gas phase molecules were used as a reference to compute the reported formation energies.

S. No.	Systems Composition	Atomization Eng. (eV)	Formation Eng. (eV)	c (Å)	Spring Const. (N/m)	Electron Affinity (eV)	Bandgap (eV)	ε_e^c	ε_{tot}^c
1	CH_2 CH_2 CH_2 CH_2	-16.332	-2.793	5.121	119.388	0.574	6.393	2.502	2.502
2	CH_2 CH_2 CH_2 GeF_2	-15.639	-7.997	5.919	86.598	1.490	5.421	2.787	4.009
3	CH_2 CH_2 CH_2 $GeCl_2$	-14.443	-3.076	6.730	52.523	3.272	3.366	2.467	3.600
4	CH_2 CH_2 CH_2 SiF_2	-16.447	-10.299	5.713	96.803	0.936	5.778	2.517	3.147
5	CH_2 CH_2 CH_2 $SiCl_2$	-15.302	-5.586	5.735	78.459	0.989	5.751	2.657	3.009
6	CH_2 CH_2 CH_2 SnF_2	-15.287	-7.241	6.633	72.733	2.903	4.085	2.421	6.237
7	CH_2 CH_2 CH_2 $SnCl_2$	-14.361	-3.403	6.840	47.156	3.115	3.327	2.533	4.255
8	CH_2 CH_2 GeF_2 GeF_2	-14.971	-13.298	6.799	64.064	2.876	4.136	3.718	7.031
9	CH_2 CH_2 $GeCl_2$ GeF_2	-14.103	-9.692	6.787	50.725	3.181	3.771	3.625	6.097
10	CH_2 CH_2 $GeCl_2$ $GeCl_2$	-13.239	-6.104	6.768	37.817	3.472	3.472	3.572	5.385

Continued on next page

*To whom correspondence should be addressed

Table 1 – continued from previous page

S. No.	System Composition	Atomization Eng. (eV)	Formation Eng. (eV)	c (Å)	Spring Const. (N/m)	Electron Affinity (eV)	Bandgap (eV)	ε_e^c	ε_{tot}^c
11	CH_2 CH_2 SiF_2 GeF_2	-15.333	-13.821	6.034	82.760	3.703	3.549	2.351	5.722
12	CH_2 CH_2 SiF_2 $GeCl_2$	-14.824	-11.648	6.589	56.452	2.335	4.463	3.095	4.673
13	CH_2 CH_2 SiF_2 SiF_2	-16.405	-17.178	6.375	75.478	1.921	4.747	2.798	4.357
14	CH_2 CH_2 $SiCl_2$ GeF_2	-14.566	-10.617	6.602	56.124	2.202	4.634	3.158	5.175
15	CH_2 CH_2 $SiCl_2$ $GeCl_2$	-13.704	-7.036	6.598	42.589	2.482	4.228	3.118	4.537
16	CH_2 CH_2 $SiCl_2$ SiF_2	-15.288	-12.579	6.398	60.970	2.045	4.651	2.831	3.851
17	CH_2 CH_2 $SiCl_2$ $SiCl_2$	-14.174	-7.988	6.430	47.831	2.127	4.596	2.868	3.696
18	CH_2 CH_2 SnF_2 GeF_2	-14.310	-11.306	6.564	72.692	3.629	2.792	3.097	12.404
19	CH_2 CH_2 SnF_2 $GeCl_2$	-13.838	-9.284	7.143	39.906	3.933	3.087	4.311	11.325
20	CH_2 CH_2 SnF_2 SiF_2	-15.220	-14.019	6.316	90.172	3.448	3.718	2.592	5.002
21	CH_2 CH_2 SnF_2 $SiCl_2$	-14.093	-9.378	6.442	55.991	3.282	3.356	2.653	5.691
22	CH_2 CH_2 SnF_2 SnF_2	-14.483	-12.649	6.634	50.568	4.443	2.980	4.271	10.462
23	CH_2 CH_2 $SnCl_2$ GeF_2	-13.402	-7.539	7.078	29.992	4.549	1.887	4.393	14.197
24	CH_2 CH_2 $SnCl_2$ $GeCl_2$	-12.547	-3.987	7.436	18.494	4.290	2.115	3.571	7.884
25	CH_2 CH_2 $SnCl_2$ SiF_2	-14.308	-10.238	6.337	57.206	4.046	2.924	2.762	4.322
26	CH_2 CH_2 $SnCl_2$ $SiCl_2$	-13.186	-5.615	6.954	24.370	3.549	2.825	2.800	3.689
27	CH_2 CH_2 $SnCl_2$ SnF_2	-13.658	-9.215	7.508	32.349	4.160	2.698	5.064	12.843
28	CH_2 CH_2 $SnCl_2$ $SnCl_2$	-12.841	-5.816	6.530	19.976	4.211	2.654	3.881	7.310
29	CH_2 GeF_2 CH_2 GeF_2	-14.920	-13.096	6.673	79.125	2.194	5.298	2.998	5.400
30	CH_2 GeF_2 CH_2 $GeCl_2$	-14.027	-9.388	6.687	66.186	2.211	4.750	3.035	4.747
31	CH_2 GeF_2 CH_2 SiF_2	-15.789	-15.645	6.473	82.387	1.801	5.595	2.799	4.701
32	CH_2 GeF_2 CH_2 $SiCl_2$	-14.613	-10.804	6.516	72.823	1.759	5.303	2.900	4.375
33	CH_2 GeF_2 CH_2 SnF_2	-14.609	-12.502	6.994	62.283	2.911	4.512	3.237	7.312
34	CH_2 GeF_2 CH_2 $SnCl_2$	-13.804	-9.147	6.988	35.223	2.831	4.132	3.182	5.750
35	CH_2 GeF_2 GeF_2 GeF_2	-14.258	-18.423	7.676	56.586	3.942	3.326	4.412	11.746
36	CH_2 GeF_2 $GeCl_2$ GeF_2	-13.402	-14.864	7.628	46.998	4.010	3.129	4.102	9.916
37	CH_2 GeF_2 SiF_2 GeF_2	-14.880	-19.980	7.454	61.181	3.304	3.815	3.702	8.073
38	CH_2 GeF_2 $SiCl_2$ GeF_2	-13.786	-15.472	7.439	49.641	3.235	3.743	3.560	6.897
39	CH_2 GeF_2 SnF_2 GeF_2	-14.033	-18.174	7.925	45.859	4.367	3.070	4.742	14.836

Continued on next page

Table 1 – continued from previous page

S. No.	System Composition	Atomization Eng. (eV)	Formation Eng. (eV)	c (Å)	Spring Const. (N/m)	Electron Affinity (eV)	Bandgap (eV)	ε_e^c	ε_{tot}^c
40	CH_2 GeF_2 $SnCl_2$ GeF_2	-13.244	-14.886	7.925	38.551	4.327	2.893	4.449	11.600
41	CH_2 $GeCl_2$ CH_2 $GeCl_2$	-13.084	-5.485	6.809	56.452	2.557	3.992	3.268	4.494
42	CH_2 $GeCl_2$ CH_2 SiF_2	-14.886	-11.896	6.503	69.929	2.105	4.825	2.862	4.174
43	CH_2 $GeCl_2$ CH_2 $SiCl_2$	-13.654	-6.835	6.660	49.536	2.400	4.168	3.149	4.198
44	CH_2 $GeCl_2$ CH_2 SnF_2	-13.718	-8.805	7.014	54.756	2.782	4.276	3.276	6.249
45	CH_2 $GeCl_2$ CH_2 $SnCl_2$	-12.882	-5.326	7.087	33.869	2.956	3.702	3.395	5.265
46	CH_2 $GeCl_2$ GeF_2 GeF_2	-13.395	-14.837	7.658	46.155	3.925	3.087	4.258	9.924
47	CH_2 $GeCl_2$ GeF_2 $GeCl_2$	-12.496	-11.107	7.686	37.864	4.365	2.739	4.320	8.422
48	CH_2 $GeCl_2$ $GeCl_2$ GeF_2	-12.524	-11.220	7.606	35.626	4.246	2.925	3.991	8.703
49	CH_2 $GeCl_2$ $GeCl_2$ $GeCl_2$	-11.662	-7.636	7.644	26.287	4.285	2.616	4.089	7.667
50	CH_2 $GeCl_2$ SiF_2 GeF_2	-13.976	-16.234	7.453	49.444	3.506	3.638	3.598	6.936
51	CH_2 $GeCl_2$ SiF_2 $GeCl_2$	-13.115	-12.654	7.500	42.592	3.524	3.305	3.690	6.854
52	CH_2 $GeCl_2$ $SiCl_2$ GeF_2	-12.905	-11.814	7.437	39.213	3.476	3.554	3.498	6.310
53	CH_2 $GeCl_2$ $SiCl_2$ $GeCl_2$	-12.042	-8.230	7.501	31.723	3.567	3.189	3.607	5.869
54	CH_2 $GeCl_2$ SnF_2 GeF_2	-13.132	-14.437	7.941	37.823	4.651	2.761	4.671	11.688
55	CH_2 $GeCl_2$ SnF_2 $GeCl_2$	-12.279	-10.890	7.964	29.785	4.698	2.442	4.710	10.227
56	CH_2 $GeCl_2$ $SnCl_2$ GeF_2	-12.385	-11.312	7.927	28.751	4.355	2.640	4.388	10.885
57	CH_2 $GeCl_2$ $SnCl_2$ $GeCl_2$	-11.513	-7.691	7.973	21.141	4.585	2.339	4.494	9.647
58	CH_2 SiF_2 CH_2 SiF_2	-16.639	-18.116	6.267	92.244	1.328	5.962	2.545	3.988
59	CH_2 SiF_2 CH_2 $SiCl_2$	-15.456	-13.251	6.328	79.297	1.234	5.793	2.682	3.908
60	CH_2 SiF_2 CH_2 SnF_2	-15.479	-15.055	6.783	63.446	2.694	4.570	3.071	5.961
61	CH_2 SiF_2 CH_2 $SnCl_2$	-14.667	-11.673	6.797	40.088	2.727	4.168	3.038	4.827
62	CH_2 SiF_2 GeF_2 GeF_2	-15.022	-20.549	7.441	59.864	3.392	3.761	3.808	9.568
63	CH_2 SiF_2 GeF_2 $GeCl_2$	-14.150	-16.928	7.446	48.643	3.519	3.436	3.712	7.342
64	CH_2 SiF_2 GeF_2 SiF_2	-15.756	-22.560	7.208	64.278	3.000	4.123	3.251	8.375
65	CH_2 SiF_2 $GeCl_2$ GeF_2	-14.171	-17.012	7.412	48.796	3.400	3.572	3.613	7.432
66	CH_2 SiF_2 $GeCl_2$ $GeCl_2$	-13.299	-13.393	7.413	39.097	3.596	3.254	3.552	6.648
67	CH_2 SiF_2 $GeCl_2$ SiF_2	-14.913	-19.051	7.196	53.591	2.945	4.020	3.151	5.797
68	CH_2 SiF_2 SiF_2 GeF_2	-15.644	-22.111	7.217	64.217	3.123	3.896	3.368	6.324

Continued on next page

Table 1 – continued from previous page

S. No.	System Composition	Atomization Eng. (eV)	Formation Eng. (eV)	c (Å)	Spring Const. (N/m)	Electron Affinity (eV)	Bandgap (eV)	ε_e^c	ε_{tot}^c
69	CH_2 SiF_2 SiF_2 $GeCl_2$	-14.776	-18.505	7.233	53.375	3.031	3.729	3.309	5.872
70	CH_2 SiF_2 SiF_2 SiF_2	-16.378	-24.119	6.974	69.059	2.850	3.937	3.003	6.149
71	CH_2 SiF_2 $SiCl_2$ GeF_2	-14.556	-17.626	7.215	52.693	3.018	3.871	3.269	6.046
72	CH_2 SiF_2 $SiCl_2$ $GeCl_2$	-13.688	-14.021	7.236	42.667	3.096	3.658	3.233	5.327
73	CH_2 SiF_2 $SiCl_2$ SiF_2	-15.301	-19.678	6.986	57.007	2.775	3.944	2.954	4.856
74	CH_2 SiF_2 SnF_2 GeF_2	-14.775	-20.214	7.677	48.668	3.699	3.540	4.061	13.731
75	CH_2 SiF_2 SnF_2 $GeCl_2$	-13.908	-16.612	7.697	40.433	3.913	3.142	4.002	11.815
76	CH_2 SiF_2 SnF_2 SiF_2	-15.225	-21.087	6.940	73.938	3.494	3.231	2.559	11.149
77	CH_2 SiF_2 $SnCl_2$ GeF_2	-13.989	-16.938	7.686	41.027	3.734	3.314	3.871	9.267
78	CH_2 SiF_2 $SnCl_2$ $GeCl_2$	-13.124	-13.342	7.723	30.731	3.943	2.960	3.858	8.435
79	CH_2 SiF_2 $SnCl_2$ SiF_2	-14.706	-18.878	7.457	44.417	3.152	3.764	3.329	7.192
80	CH_2 $SiCl_2$ CH_2 $SiCl_2$	-14.206	-8.115	6.521	70.474	1.562	5.027	2.980	3.800
81	CH_2 $SiCl_2$ CH_2 SnF_2	-14.313	-10.257	6.826	56.678	2.624	4.447	3.153	5.671
82	CH_2 $SiCl_2$ CH_2 $SnCl_2$	-13.467	-6.739	6.925	37.900	2.913	3.796	3.299	4.848
83	CH_2 $SiCl_2$ GeF_2 GeF_2	-13.898	-15.919	7.450	49.524	3.373	3.565	3.786	8.398
84	CH_2 $SiCl_2$ GeF_2 $GeCl_2$	-13.007	-12.223	7.495	41.202	3.731	3.111	3.873	8.693
85	CH_2 $SiCl_2$ GeF_2 SiF_2	-14.628	-17.914	7.230	54.609	2.904	3.997	3.266	7.675
86	CH_2 $SiCl_2$ GeF_2 $SiCl_2$	-13.454	-13.083	7.319	46.081	3.284	3.642	3.440	6.036
87	CH_2 $SiCl_2$ $GeCl_2$ GeF_2	-13.027	-12.304	7.423	38.696	3.723	3.367	3.630	7.390
88	CH_2 $SiCl_2$ $GeCl_2$ $GeCl_2$	-12.157	-8.688	7.484	29.960	3.905	2.933	3.748	6.528
89	CH_2 $SiCl_2$ $GeCl_2$ SiF_2	-13.759	-14.305	7.236	42.525	3.023	3.937	3.194	5.667
90	CH_2 $SiCl_2$ $GeCl_2$ $SiCl_2$	-12.633	-9.667	7.327	34.373	3.284	3.488	3.368	5.475
91	CH_2 $SiCl_2$ SiF_2 GeF_2	-14.475	-17.301	7.239	53.414	3.169	3.785	3.343	6.104
92	CH_2 $SiCl_2$ SiF_2 $GeCl_2$	-13.607	-13.693	7.317	45.688	3.268	3.441	3.447	5.739
93	CH_2 $SiCl_2$ SiF_2 SiF_2	-15.196	-19.257	7.016	57.956	2.851	3.856	3.016	5.449
94	CH_2 $SiCl_2$ SiF_2 $SiCl_2$	-14.112	-14.789	7.138	50.879	2.930	3.599	3.180	5.539
95	CH_2 $SiCl_2$ $SiCl_2$ GeF_2	-13.434	-13.004	7.249	42.940	3.001	3.749	3.288	5.774
96	CH_2 $SiCl_2$ $SiCl_2$ $GeCl_2$	-12.547	-9.320	7.346	34.230	3.339	3.335	3.412	5.240
97	CH_2 $SiCl_2$ $SiCl_2$ SiF_2	-14.179	-15.057	7.051	46.128	2.755	3.873	2.991	4.762

Continued on next page

Table 1 – continued from previous page

S. No.	System Composition	Atomization Eng. (eV)	Formation Eng. (eV)	c (Å)	Spring Const. (N/m)	Electron Affinity (eV)	Bandgap (eV)	ε_e^c	ε_{tot}^c
98	CH_2 $SiCl_2$ $SiCl_2$ $SiCl_2$	-13.031	-10.332	7.205	38.797	2.993	3.586	3.169	4.615
99	CH_2 $SiCl_2$ SnF_2 GeF_2	-13.660	-15.621	7.715	40.337	3.819	3.250	4.096	13.130
100	CH_2 $SiCl_2$ SnF_2 $GeCl_2$	-12.779	-11.963	7.766	33.489	4.140	2.798	4.174	9.601
101	CH_2 $SiCl_2$ SnF_2 SiF_2	-14.365	-17.512	7.465	44.024	3.138	3.796	3.454	9.162
102	CH_2 $SiCl_2$ SnF_2 $SiCl_2$	-13.235	-12.860	7.564	36.483	3.514	3.339	3.628	9.142
103	CH_2 $SiCl_2$ $SnCl_2$ GeF_2	-12.874	-12.341	7.733	31.461	3.871	3.044	3.949	8.927
104	CH_2 $SiCl_2$ $SnCl_2$ $GeCl_2$	-11.994	-8.687	7.819	23.154	4.221	2.635	4.079	7.972
105	CH_2 $SiCl_2$ $SnCl_2$ SiF_2	-13.588	-14.272	7.516	33.607	3.257	3.535	3.395	7.085
106	CH_2 $SiCl_2$ $SnCl_2$ $SiCl_2$	-12.455	-9.607	7.658	26.309	3.668	3.097	3.608	6.696
107	CH_2 SnF_2 CH_2 SnF_2	-14.280	-11.838	7.302	59.068	3.221	4.136	3.439	9.184
108	CH_2 SnF_2 CH_2 $SnCl_2$	-13.480	-8.506	7.317	47.802	3.117	3.910	3.395	7.346
109	CH_2 SnF_2 GeF_2 GeF_2	-13.986	-17.985	7.926	35.519	4.331	3.068	4.723	17.386
110	CH_2 SnF_2 GeF_2 $GeCl_2$	-13.131	-14.432	7.942	37.161	4.239	2.840	4.675	15.646
111	CH_2 SnF_2 GeF_2 SiF_2	-14.748	-20.106	7.680	40.149	3.827	3.396	4.155	11.500
112	CH_2 SnF_2 GeF_2 $SiCl_2$	-13.636	-15.525	7.709	36.393	3.800	3.202	4.196	11.959
113	CH_2 SnF_2 GeF_2 SnF_2	-13.713	-17.547	8.250	43.442	4.595	2.774	5.229	21.613
114	CH_2 SnF_2 $GeCl_2$ GeF_2	-13.136	-14.450	7.900	36.547	4.390	2.812	4.433	14.291
115	CH_2 SnF_2 $GeCl_2$ $GeCl_2$	-12.282	-10.903	7.929	28.675	4.339	2.636	4.432	11.775
116	CH_2 SnF_2 $GeCl_2$ SiF_2	-13.905	-16.602	7.679	33.652	3.957	3.108	3.972	10.783
117	CH_2 SnF_2 $GeCl_2$ $SiCl_2$	-12.793	-12.017	7.725	30.397	3.954	2.934	4.049	10.533
118	CH_2 SnF_2 $GeCl_2$ SnF_2	-12.868	-14.031	8.241	35.570	4.654	2.531	4.910	17.737
119	CH_2 SnF_2 SiF_2 GeF_2	-14.845	-20.495	7.690	41.216	3.424	3.623	3.939	10.551
120	CH_2 SnF_2 SiF_2 $GeCl_2$	-13.680	-15.702	7.707	37.090	3.598	3.464	3.884	9.210
121	CH_2 SnF_2 SiF_2 SiF_2	-15.254	-21.205	7.427	43.901	3.393	3.684	3.650	8.092
122	CH_2 SnF_2 SiF_2 $SiCl_2$	-14.174	-16.751	7.466	42.412	3.294	3.587	3.637	8.725
123	CH_2 SnF_2 SiF_2 SnF_2	-14.208	-18.597	7.992	47.277	3.888	3.370	4.270	14.685
124	CH_2 SnF_2 $SiCl_2$ GeF_2	-13.449	-14.777	7.695	39.620	3.778	3.442	3.794	8.929
125	CH_2 SnF_2 $SiCl_2$ $GeCl_2$	-12.620	-11.328	7.733	32.494	3.705	3.263	3.794	7.811
126	CH_2 SnF_2 $SiCl_2$ SiF_2	-14.206	-16.878	7.457	40.966	3.423	3.551	3.542	9.181

Continued on next page

Table 1 – continued from previous page

S. No.	System Composition	Atomization Eng. (eV)	Formation Eng. (eV)	c (Å)	Spring Const. (N/m)	Electron Affinity (eV)	Bandgap (eV)	ε_e^c	ε_{tot}^c
127	CH_2 SnF_2 $SiCl_2$ $SiCl_2$	-13.124	-12.415	7.518	33.954	3.415	3.416	3.577	7.258
128	CH_2 SnF_2 $SiCl_2$ SnF_2	-13.156	-14.257	8.012	38.008	3.967	3.144	4.113	12.108
129	CH_2 SnF_2 SnF_2 GeF_2	-13.687	-17.443	8.133	36.344	4.798	2.880	4.969	26.035
130	CH_2 SnF_2 SnF_2 $GeCl_2$	-12.864	-14.014	8.176	30.293	4.721	2.616	4.994	18.353
131	CH_2 SnF_2 SnF_2 SiF_2	-14.506	-19.792	7.856	34.048	4.129	3.165	4.344	16.692
132	CH_2 SnF_2 SnF_2 $SiCl_2$	-13.406	-15.256	7.927	31.696	4.108	2.982	4.432	18.173
133	CH_2 SnF_2 SnF_2 SnF_2	-13.504	-17.364	8.425	37.711	4.839	2.649	5.373	46.935
134	CH_2 SnF_2 $SnCl_2$ GeF_2	-12.986	-14.504	8.161	30.581	4.595	2.682	4.710	17.853
135	CH_2 SnF_2 $SnCl_2$ $GeCl_2$	-12.138	-10.979	8.235	25.579	4.584	2.451	4.831	14.457
136	CH_2 SnF_2 $SnCl_2$ SiF_2	-13.732	-16.559	7.905	29.954	4.115	2.981	4.158	16.621
137	CH_2 SnF_2 $SnCl_2$ $SiCl_2$	-12.629	-12.016	7.999	25.607	4.177	2.762	4.333	11.361
138	CH_2 SnF_2 $SnCl_2$ SnF_2	-12.727	-14.119	8.460	31.300	4.798	2.479	5.099	26.252
139	CH_2 $SnCl_2$ CH_2 $SnCl_2$	-12.658	-5.085	7.403	39.872	3.250	3.542	3.520	6.100
140	CH_2 $SnCl_2$ GeF_2 GeF_2	-13.202	-14.717	7.939	32.370	4.248	2.860	4.540	14.382
141	CH_2 $SnCl_2$ GeF_2 $GeCl_2$	-12.330	-11.095	7.989	28.298	4.341	2.581	4.647	11.044
142	CH_2 $SnCl_2$ GeF_2 SiF_2	-13.955	-16.801	7.708	35.790	3.878	3.140	4.017	9.317
143	CH_2 $SnCl_2$ GeF_2 $SiCl_2$	-12.830	-12.166	7.778	31.611	4.008	2.875	4.192	8.715
144	CH_2 $SnCl_2$ GeF_2 SnF_2	-12.933	-14.293	8.267	32.145	4.462	2.632	5.021	19.525
145	CH_2 $SnCl_2$ GeF_2 $SnCl_2$	-12.139	-10.984	8.335	26.478	4.537	2.400	5.050	13.681
146	CH_2 $SnCl_2$ $GeCl_2$ GeF_2	-12.350	-11.175	7.917	28.922	4.324	2.666	4.298	10.148
147	CH_2 $SnCl_2$ $GeCl_2$ $GeCl_2$	-11.482	-7.570	7.981	22.235	4.471	2.405	4.452	9.782
148	CH_2 $SnCl_2$ $GeCl_2$ SiF_2	-13.111	-13.290	7.714	31.091	3.967	2.900	3.877	8.006
149	CH_2 $SnCl_2$ $GeCl_2$ $SiCl_2$	-11.985	-8.654	7.804	23.517	4.180	2.645	4.095	8.055
150	CH_2 $SnCl_2$ $GeCl_2$ SnF_2	-12.086	-10.771	8.267	27.515	4.581	2.411	4.780	13.973
151	CH_2 $SnCl_2$ $GeCl_2$ $SnCl_2$	-11.292	-7.460	8.350	19.323	4.681	2.210	4.858	12.383
152	CH_2 $SnCl_2$ SiF_2 GeF_2	-13.804	-16.196	7.709	36.031	3.441	3.416	3.805	9.841
153	CH_2 $SnCl_2$ SiF_2 $GeCl_2$	-12.936	-12.590	7.786	33.107	3.541	3.138	3.927	7.876
154	CH_2 $SnCl_2$ SiF_2 SiF_2	-14.561	-18.295	7.472	38.884	3.246	3.488	3.539	7.109
155	CH_2 $SnCl_2$ SiF_2 $SiCl_2$	-13.440	-13.679	7.572	32.313	3.378	3.264	3.699	6.879

Continued on next page

Table 1 – continued from previous page

S. No.	System Composition	Atomization Eng. (eV)	Formation Eng. (eV)	c (Å)	Spring Const. (N/m)	Electron Affinity (eV)	Bandgap (eV)	ε_e^c	ε_{tot}^c
156	CH_2 $SnCl_2$ SiF_2 SnF_2	-13.514	-15.689	8.032	34.977	3.637	3.197	4.160	12.590
157	CH_2 $SnCl_2$ SiF_2 $SnCl_2$	-12.839	-12.856	8.115	30.235	3.761	2.947	4.198	10.465
158	CH_2 $SnCl_2$ $SiCl_2$ GeF_2	-12.718	-11.718	7.724	31.893	3.584	3.233	3.709	7.854
159	CH_2 $SnCl_2$ $SiCl_2$ $GeCl_2$	-11.868	-8.183	7.829	24.802	3.797	2.938	3.869	7.050
160	CH_2 $SnCl_2$ $SiCl_2$ SiF_2	-13.484	-13.854	7.516	33.500	3.407	3.314	3.478	6.347
161	CH_2 $SnCl_2$ $SiCl_2$ $SiCl_2$	-12.359	-9.222	7.660	25.568	3.615	3.039	3.681	6.142
162	CH_2 $SnCl_2$ $SiCl_2$ SnF_2	-12.436	-11.244	8.068	29.895	3.846	2.981	4.054	10.278
163	CH_2 $SnCl_2$ $SiCl_2$ $SnCl_2$	-11.637	-7.911	8.187	21.925	4.027	2.711	4.164	8.899
164	CH_2 $SnCl_2$ SnF_2 GeF_2	-12.932	-14.288	8.162	30.773	4.686	2.651	4.814	16.440
165	CH_2 $SnCl_2$ SnF_2 $GeCl_2$	-12.093	-10.797	8.240	24.574	4.755	2.359	4.993	17.291
166	CH_2 $SnCl_2$ SnF_2 SiF_2	-13.648	-16.225	7.923	30.200	4.213	2.941	4.239	15.408
167	CH_2 $SnCl_2$ SnF_2 $SiCl_2$	-12.563	-11.752	8.018	24.735	4.325	2.665	4.461	15.522
168	CH_2 $SnCl_2$ SnF_2 SnF_2	-12.669	-13.886	8.477	27.824	4.830	2.477	5.273	26.640
169	CH_2 $SnCl_2$ SnF_2 $SnCl_2$	-11.907	-10.705	8.578	20.062	4.865	2.252	5.370	18.882
170	CH_2 $SnCl_2$ $SnCl_2$ GeF_2	-12.171	-11.110	8.211	23.148	4.622	2.497	4.651	12.724
171	CH_2 $SnCl_2$ $SnCl_2$ $GeCl_2$	-11.335	-7.630	8.336	17.001	4.754	2.191	4.926	11.598
172	CH_2 $SnCl_2$ $SnCl_2$ SiF_2	-12.900	-13.100	7.983	25.628	4.214	2.751	4.124	10.569
173	CH_2 $SnCl_2$ $SnCl_2$ $SiCl_2$	-11.819	-8.643	8.173	17.943	4.465	2.415	4.520	9.889
174	CH_2 $SnCl_2$ $SnCl_2$ SnF_2	-11.914	-10.734	8.543	22.300	4.813	2.312	5.105	17.139
175	CH_2 $SnCl_2$ $SnCl_2$ $SnCl_2$	-11.154	-7.562	8.703	15.242	4.909	2.051	5.367	14.355
